# Repeated elevated plus maze trials as a measure for tracking within-subjects behavioral performance in rats (*Rattus norvegicus*)

**DOI:** 10.1371/journal.pone.0207804

**Published:** 2018-11-26

**Authors:** Andrew J. Schrader, Rachel M. Taylor, Emily G. Lowery-Gionta, Nicole L. T. Moore

**Affiliations:** 1 Veterinary Services Program, Walter Reed Army Institute of Research, Silver Spring, MD; 2 Performance Assessment and Chemical Evaluation (PACE) Laboratory, Department of Behavioral Biology, Center for Military Psychiatry and Neuroscience, Walter Reed Army Institute of Research, Silver Spring, MD; Technion Israel Institute of Technology, ISRAEL

## Abstract

The elevated plus maze (EPM) is routinely used in neuroscience research to evaluate emotional behavior in rodents by measuring general exploratory performance and avoidance of the aversive open arms of the maze. According to standard practice, behavior on the EPM is evaluated during a single trial to avoid the possibility of habituation to the apparatus that would result in lost sensitivity of key outcome measures. However, this possibility has not been systematically evaluated across repeated trials or across different environmental conditions. In the current study, we assessed within-subject behavior on the EPM in adult male rats over thirteen trials (tested twice weekly) repeated under identical conditions. We also assessed within-subject behavior on the EPM in adult male rats under dim (1 lux in the closed arm) and lit (246 lux in the closed arm) environmental conditions. We found that measures of general performance (basic movements and total distanced travelled throughout the maze) were stable across repeated trials and environmental conditions. We found that measures of open arm avoidance (distance travelled in, time spent in and entries in to the open arm) varied across trials and environmental conditions and were sensitive to the lighting conditions of the initial test. Though measures of open arm avoidance did show a linear trend indicative of habituation across repeated trials, this effect was variable across trials. Notably, preference for the open arm over the closed arm (measured as % of time spent in the open arm) assessed among individual animals occurred rarely and was never observed on the group level across the thirteen repeated trials. Together, these data demonstrate that measures of general performance such as basic movements and total distance traveled are robust to repeated testing and changing environmental lighting conditions. In contrast, measures of open arm avoidance show habituation with repeated testing and are sensitive to changing environmental lighting conditions. Based on these results, we suggest that within-subjects repeated testing on the EPM is valid in well-controlled studies that include an untreated control group to account for inter-trial variability and habituation.

## Introduction

The elevated plus maze (EPM) is a preclinical test of avoidance behavior in rodents that has been commonly used to assess the effects of experimental manipulations on emotional behavioral performance [1]. Since its first emergence to the field of neurobehavioral research thirty years ago, it has been integral for understanding the biological basis of emotionality [2]. The test relies on an approach-avoidance conflict that weighs rodents’ thigmotaxic preferences for familiar, protected and enclosed spaces against their motivation to explore unfamiliar, exposed or open spaces in search of survival means like food, mates or shelter [3].

Evaluation of a subject’s emotional behavioral performance is based upon shifts in the approach-avoidance conflict that are revealed by changes in where an animal distributes its time on the apparatus. The EPM is a four-armed maze that is configured as a “plus” sign and elevated away from the floor. Two of the arms are enclosed by walls (the “closed arms”) and two of the arms are open (the “open arms”). Typically, rodents show avoidance of the open arms and a predilection for the more protected or “safe” closed arms analogous to the rodents’ outer-perimeter preference in the open-field test and dark-space preference in the light/dark test. Relative distributions of time and behavioral performance in the open versus closed arms are used to interpret open arm avoidance, which translates to clinically relevant negative emotional states like aversion, hypervigilance, risk tolerance and anxiety [4]. As such, shifts in behavioral performance that reflect an overall increase in open arm avoidance are interpreted as increased anxiety and reduced risk tolerance. These interpretations are based on pharmacological validations of the EPM, as compounds that attenuate anxiety in people produce reliable reductions in open arm avoidance in rodent subjects on the EPM. Additionally, the translatability and validity of the EPM was recently evaluated in people, who reported feelings of anxiety and aversion when experiencing a virtual reality simulation of elevated open arms [5].

Alongside measures of open arm avoidance, general performance can be evaluated in the EPM [6]. Typically, measures of general performance are related to locomotor activity–the total basic movements performed or total distance travelled in the maze and entries made in to both arms. Experimentally, these variables are most often treated as controls for confounding locomotor effects of a manipulation. For example, reductions in general performance variables could indicate malaise, locomotor incapacity or sedative effects [7]. However, in addition to these indications of somatic distress, general performance variables may also provide important insights to emotional status as negative emotional states are often accompanied by reduced movement and exploration.

Both open arm avoidance and general performance measures on the EPM are affected by experimental manipulations, including exposure to stress or psychoactive pharmacological compounds [2, 8–10]. As such, the EPM has been a useful tool for assessing pharmacological compounds for their anxiolytic potential [1], examining the impact of environmental stressors or drugs of abuse on emotional behavior [11, 12] and investigating the neurobiological underpinnings of emotionality [13]. Typically, the effects of these manipulations are examined during a single EPM exposure, thus limiting the practical use of the EPM to a single trial. Indeed, trends of behavioral performance on the EPM over time are rarely evaluated or published. Though data gained from a single trial is certainly informative, longitudinal evaluations would allow the examination of many important experimental questions.

The practice of assessing behavioral performance during a single trial of EPM is based on observations of one-trial tolerance to the effects of benzodiazepines on open arm avoidance, and the possibility that habituation to the apparatus diminishes open arm avoidance over repeated trials. One-trial tolerance is a phenomenon characterized by File and colleagues based on observations that the anxiolytic effects of benzodiazepines, revealed by reductions in open arm avoidance, decrease across multiple EPM trials [2, 14–16]. Follow-up studies have consistently demonstrated within-subject reductions in the effects of benzodiazepines on open arm avoidance between the initial and second trials [17–19]. The authors of these studies postulate that the reduction in benzodiazepine efficacy stems from learning that occurs during the initial EPM trial, in which behavioral performance is driven by a “phobic anxiety state” stemming from both novelty of the maze and a fear of heights [20]. By the second trial, those “anxiety states” driven by novelty resolve as the subject habituates to the maze but those driven by fear of heights do not. According to this hypothesis, the factors that drive behavioral performance differ across repeated EPM trials and thus may result in habituated open arm avoidance or general performance measures, precluding repeated testing due to floor or ceiling effects. While many studies that have provided an evaluation of the one-trial tolerance phenomenon as it relates to EPM behavioral performance over repeated trials, few studies provide a systematic evaluation and characterization of overall EPM behavioral performance over time and without benzodiazepine treatment as a factor [21–26]. Of these studies, many evaluated the effect of repeated EPM trials on open arm avoidance metrics without an accompanying characterization of general locomotor performance.

In the current study, we evaluated the behavioral performance of experimentally-naïve rats on the EPM over thirteen trials conducted under identical environmental conditions. We hypothesized that 1) measures of general performance (as measured by basic movements and total distance travelled) would be stable across trials and 2) measures of open arm avoidance (as measured by time spent in, distance travelled in and entries made to the open arm) would habituate over trials but not to the point that consistent open arm preference would be observed. Additionally, we probed the stability of these measures across environmental conditions by assessing behavioral performance in a brightly lit versus dim EPM. We hypothesized that measures showing stability across time would also be stable across lighting conditions.

## Materials and methods

### Subjects

A total of 21 adult male Sprague-Dawley rats (Charles River Laboratories, Wilmington, MA) were housed on a 12 h:12 h light:dark schedule with lights on at 7 AM. Rats were acclimated to the facility for one week, and then handled once daily for four days by both male and female researchers prior to experimental manipulation. Rats were singly housed with mild food restriction to maintain a maximum weight of 350g, in concordance with previous studies in our laboratory to promote exploratory drive during behavioral testing. The food restriction protocol provided each rat with a daily food allowance equivalent to or greater than their minimum energy requirements; caloric intake was calibrated by routine (daily Monday through Friday) weight checks. Research was conducted under an animal use protocol approved by Walter Reed Army Institute of Research’s Institutional Animal Care and Use Committee in an AAALAC accredited facility. All procedures are in compliance with the Animal Welfare Act and other federal statutes and regulations relating to animals and experiments involving animals and adheres to principles stated in the Guide for the Care and Use of Laboratory Animals, NRC Publication, 2011 edition. The 3 R’s of animal research were considered and used in this protocol and environmental enrichment was provided for the duration of the study. At the conclusion of this study, all animals were either humanely euthanized with carbon dioxide gas/bilateral thoracotomy in accordance with the 2013 AVMA Guidelines for Euthanasia of Animals or transferred to a facility-wide animal use training protocol.

### Experimental design

In Experiment 1, eight adult male rats were evaluated on the elevated plus maze over 13 sessions conducted twice weekly beginning approximately 3 hours in to the light phase. All testing was conducted during the light phase of the light/dark cycle. Each session lasted 15 minutes and was conducted under standard lights-on conditions, with high contrast between the open and closed arms (lux: 999 for open arms, 246 for closed arms, 826 for intersection). The EPM (Kinder Scientific, Poway, CA) consisted of four tinted plastic arms (length: 50 cm; width: 10 cm) in a “plus” configuration, elevated 80 cm above the floor that was located in a separate room from the housing room. Closed arms had walls 40 cm in height and open arms had no walls. Each test began by placing the rat facing the intersection of the maze. The EPM was cleaned immediately after each test to mitigate the possibility of introducing pheromonal cues. Exploratory behavior on the maze was measured using a Kinder Scientific automated EPM software and photobeam tracking. Basic movements, total distance travelled, open/closed arm entries, open/closed arm time, and open/closed arm distance were used as the dependent measures. Basic movements were defined as the count of consecutive photobeam interruptions.

In Experiment 2, 13 adult male rats were evaluated on the EPM over two trials, each lasting 5 minutes, using a crossover study design. Testing began approximately 3 hours in to the light phase; all testing was conducted during the light phase of the light/dark cycle. The first trial was conducted under dim lighting conditions (lux: 7 for open arms, 1 for closed arms, 2 for intersection) for 6 rats (Dim First group) and standard lights-on conditions described above for 7 rats (Lit First group). For the second trial, conditions for each group were reversed. The EPM apparatus and dependent measures were the same as described earlier.

### Statistical analysis

All statistical analyses were performed using GraphPad Prism 7.0 software (La Jolla, CA). For Experiment 1, data for each dependent measure were analyzed using a repeated-measures one-way ANOVA (sphericity not assumed; Greenhouse-Geisser correction applied). Significant ANOVA results were further explored using Dunnett’s test for multiple comparisons as *post-hoc* analyses (also with Greenhouse-Geisser correction applied) to evaluate changes in behavioral performance between trials relative to the first trial. To assess trends in each dependent measure across time, linear regression analyses were used to determine lines of best fit. A Spearman’s correlation (two-tailed) was used to evaluate the correlation between basic movements and total distance travelled for each trial. For Experiment 2, data were analyzed using a repeated-measures two-way ANOVA with lighting conditions and trial order as factors. Sidak’s test was used for *post-hoc* analyses. Statistical significance was set at an alpha-level of less than 0.05. All data are presented as means ± standard errors of the mean.

## Results

### Experiment 1: Repeated elevated plus maze trials

**Fig 1:**
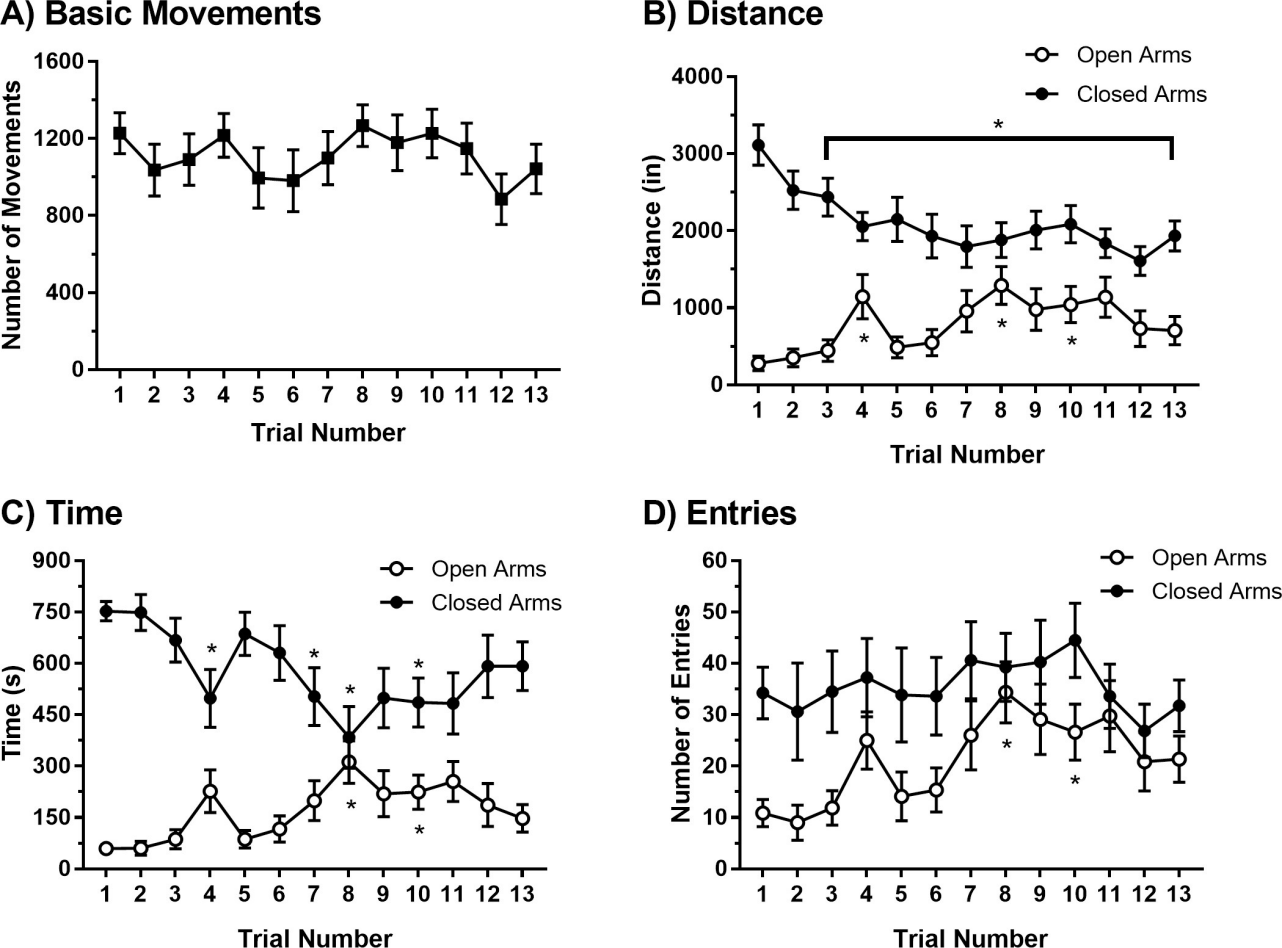
Behavior on the EPM varies across 13 trials relative to the first trial. Basic movements (A), open/closed arm distance (B), open/closed arm time (C), and open/closed arm entries (D) across 13 repeated EPM trials. Data are presented as mean and standard error (N = 8). Trials that significantly differ from Trial 1 are indicated by an asterisk (*).

**Fig 2:**
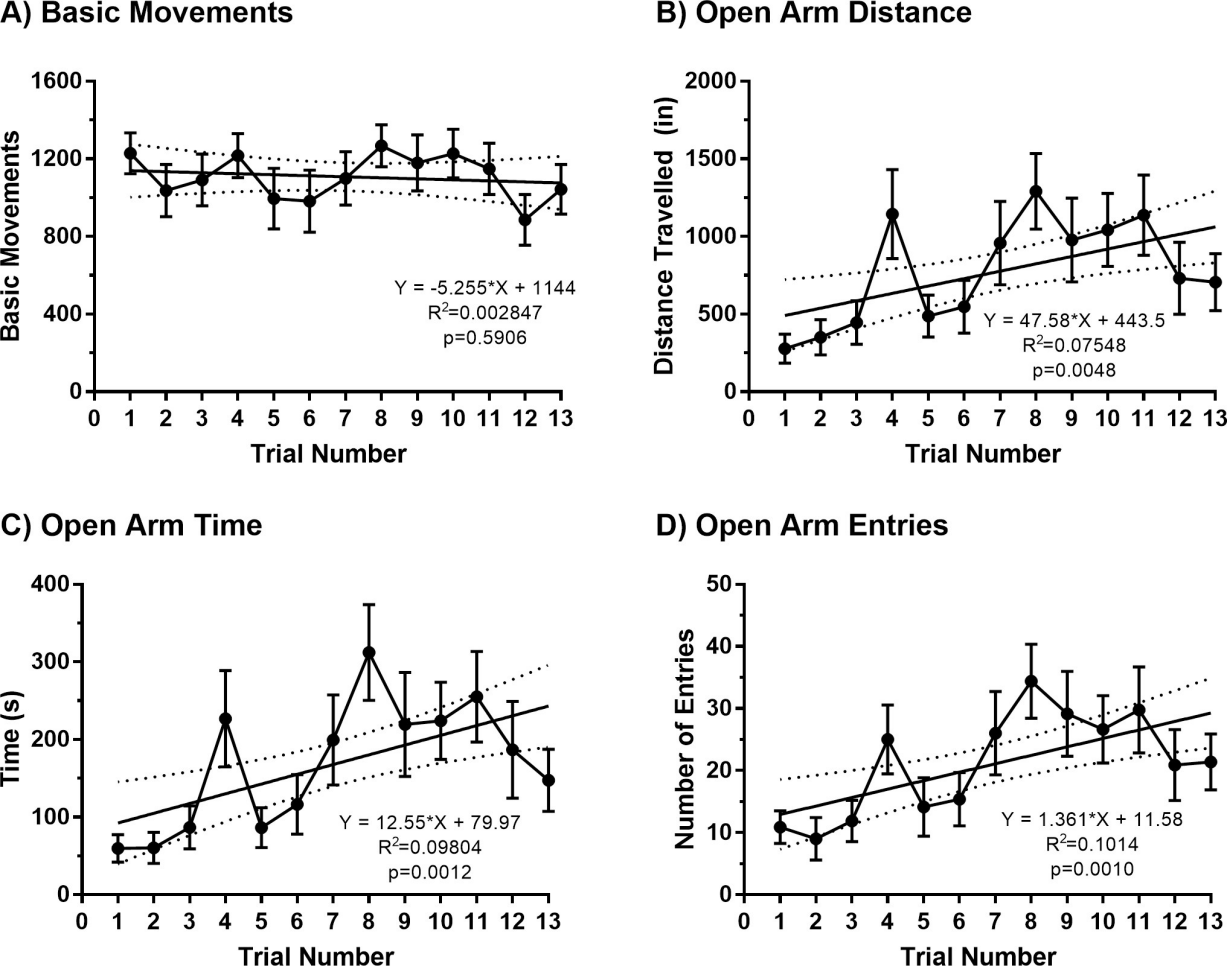
Linear trends for general performance and open arm measures across repeated EPM trials. Lines of best fit for basic movements (A), open arm distance (B), open arm time (C), and open arm entries (D) cross 13 repeated EPM trials (Experiment 1). Data are presented as mean and standard error (N = 8), with the equation and p-value for each analysis shown on the graph.

**Fig 3:**
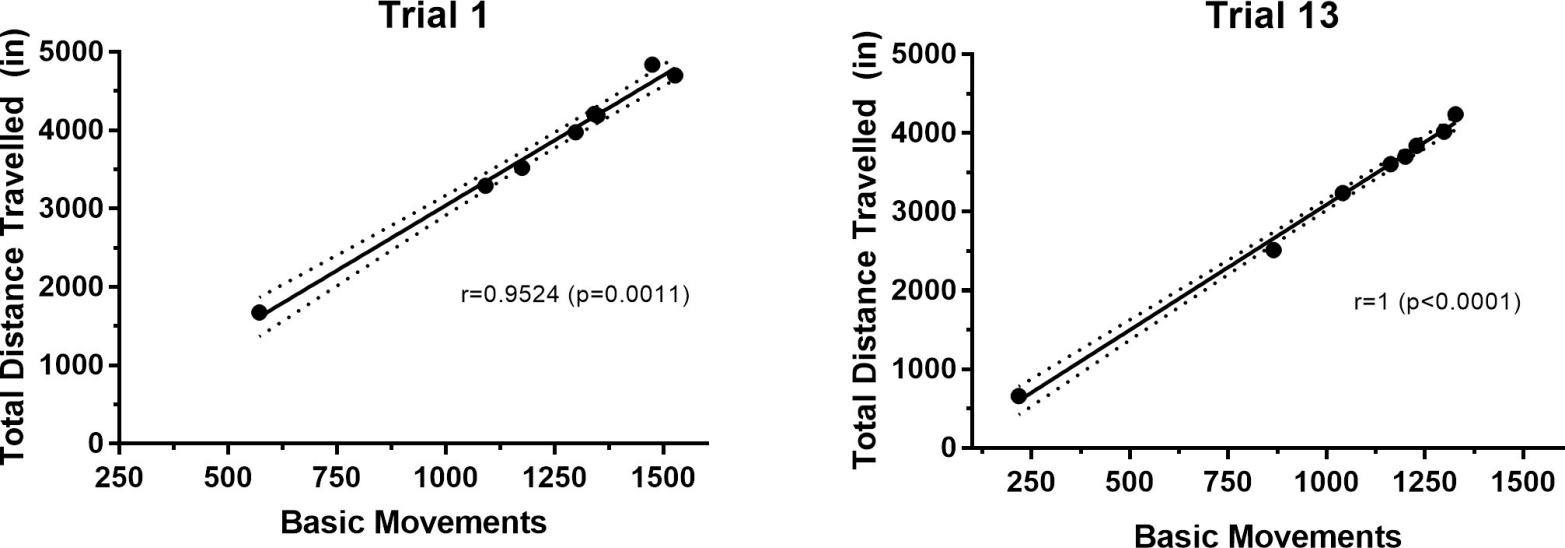
Correlation of basic movements and total distance travelled on the EPM. Basic movements and total distance travelled are highly correlated at the first trial (left-side panel) as well as the final, 13^th^ trial (right-side panel). Data are presented as the raw value for basic movements and total distance travelled, with the calculated Spearman’s correlation coefficient and p-value shown for the first and last trial.

We first evaluated the effect of 13 repeated EPM trials on behavioral performance under standard lighting conditions (see Figs 1 and 2). Table 1 provides the specific results of each repeated-measures one-way ANOVA and *post-hoc* test used to evaluate statistical significance for each outcome measure and trial. As shown in Figs 1A and 2A, basic movements were fairly stable over the 13 trials. The results of a repeated-measures one-way ANOVA showed no significant effect of time across trials [F(2.82, 19.7) = 2.98, p = 0.059]. Likewise, a linear regression analysis performed on these data showed that the line of best fit did not differ significantly from zero (Equation: y = -5.255x + 1144; F (1, 102) = 0.2912, p = 0.5906), indicating that time was not a predictive factor in the number of basic movements performed across trials. The results of a Spearman’s correlation analysis showed that measures of basic movements and total distance travelled at the first trial as well as the final trial were highly correlated (Fig 3; Trial 1: r = 0.9524, p = 0.0011; Trial 13: r = 1, p<0.0001); therefore, basic movements and total distance travelled on the EPM are related general performance measurements. In contrast to measures of general performance, measures of open arm avoidance showed less stability over repeated trials. As shown in Figs 1B–1D and 2B–2D, the results of repeated-measures one-way ANOVA revealed a significant effect of time across trials for distance travelled in the open arm [F(3.74, 26.2) = 5.59, p = 0.003], time spent in the open arm [F(3.68, 25.8) = 5.94, p = 0.002] and entries made to the open arm [F(3.75, 26.3) = 5.56, p = 0.003]. *Post-hoc* analyses comparing each of these variables in the first trial to all other trials are shown in Table 1. Linear regression analyses performed on each open arm variable revealed significant linear trends across trials. Specifically, the lines of best fit deviated significantly from zero for distance travelled in the open arm (Equation: y = 47.58x + 443.5; F (1, 102) = 8.328, p = 0.0048), time spent in the open arm (Equation: y = 12.55x + 79.97; F(1, 102) = 11.09, p = 0.0012), and entries made in to the open arm (Equation: y = 1.361x + 11.58; F(1, 102) = 11.51, p = 0.0010). Consistent with these observations, repeated-measures one-way ANOVAs revealed significant main effects of time for distance travelled in the closed arm [F(3.61, 25.3) = 8.01, p<0.001] and time spent in the closed arm [F(3.24, 22.9) = 5.97, p = 0.003]. However, a significant main effect of time was not observed for closed arm entries [F(3.14, 22.0) = 0.987, p = 0.420]. *Post-hoc* analyses revealed specific differences between Trial 1 and select subsequent trials, as summarized in Table 1. Linear regression analyses performed on closed arm measures revealed significant linear trends. Specifically, the line of best fit deviated significantly from zero for distance travelled in the closed arm (Equation: y = -78.58x + 2655; F(1, 102) = 19.52, p<0.0001) and time spent in the closed arm (Equation: y = -17.29x + 700; F(1, 102) = 8.93, p = 0.0035), but not closed arm entries (Equation: y = 0.0158x + 35.35; F(1, 102) = 0.0009103, p = 0.9760).

**Table 1:** Statistical results of raw data from Experiment 1. Repeated-measures one-way ANOVA and Dunnett’s *post-hoc* test results showing main effects of time across repeated trials for basic movements, open/closed arm distance, open/closed arm time, and open/closed arm entries. Trials not significantly different from the first trial are reported as “ns”; trials that differ significantly from the first trial are reported with an associated p-value.

		Basic Movements	Open Arm Distance	Open Arm Time	Open Arm Entries	Closed Arm Distance	Closed Arm Time	Closed Arm Entries
**ANOVA p-value**		0.059	0.003	0.002	0.003	0.0004	0.003	0.420
**Dunnett's Multiple Comparisons**	**1 vs. 2**	ns	ns	ns	ns	ns	ns	ns
	**1 vs. 3**	ns	ns	ns	ns	0.002	ns	ns
	**1 vs. 4**	ns	0.035	ns	ns	0.022	0.026	ns
	**1 vs. 5**	ns	ns	ns	ns	<0.001	ns	ns
	**1 vs. 6**	ns	ns	ns	ns	0.002	ns	ns
	**1 vs. 7**	ns	ns	ns	ns	<0.001	0.043	ns
	**1 vs. 8**	ns	0.014	0.017	0.025	0.009	0.013	ns
	**1 vs. 9**	ns	ns	ns	ns	<0.001	ns	ns
	**1 vs. 10**	ns	0.042	0.044	0.041	0.020	0.017	ns
	**1 vs. 11**	ns	ns	ns	ns	<0.001	ns	ns
	**1 vs. 12**	ns	ns	ns	ns	0.003	ns	ns
	**1 vs. 13**	ns	ns	ns	ns	0.002	ns	ns

**Fig 4:**
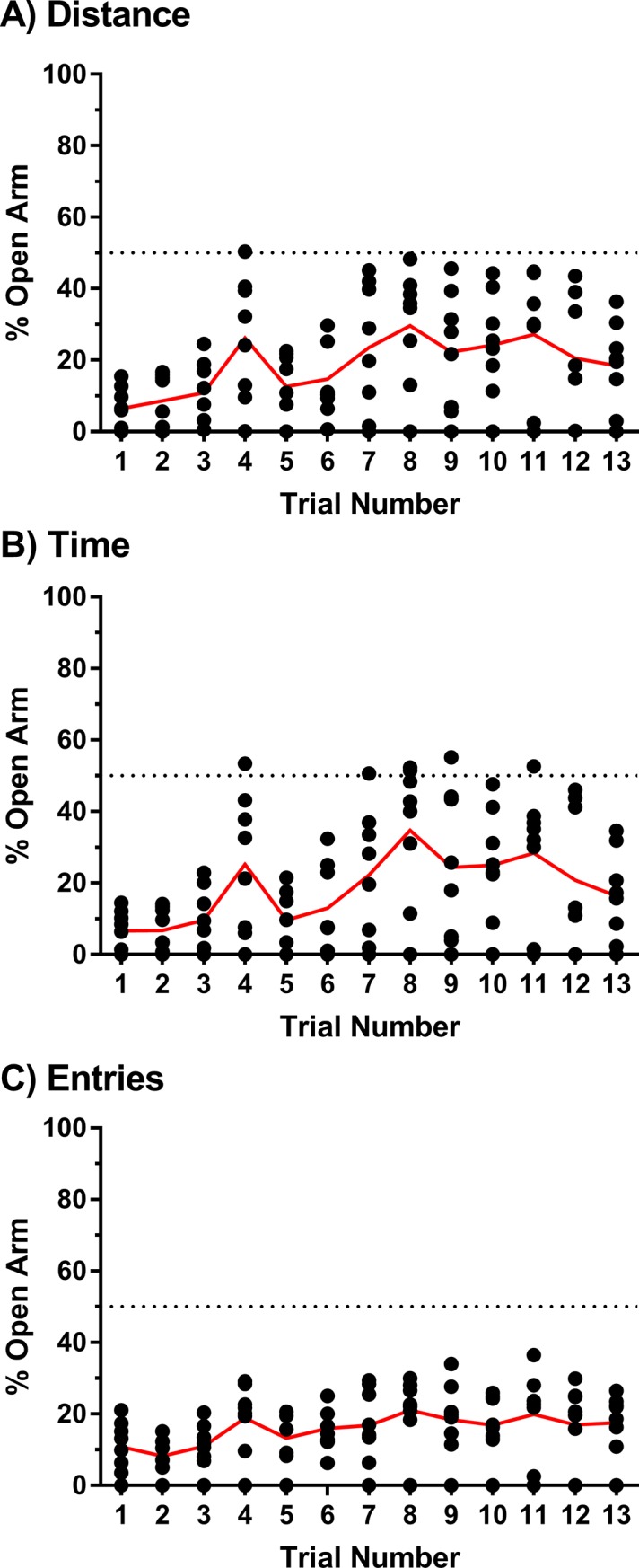
Open arm preference across repeated EPM trials. Open arm avoidance measures presented as percentages of total distance (A), total time (B), and total entries (C). Values were computed by dividing raw open arm measurements by raw total measurements. Data are presented as single subject points with the group mean (N = 8) denoted by a red line.

Additionally, measures of open arm avoidance were calculated as a percentage of the total distance travelled, total time spent, or total number of entries made (total of each was the sum of open arm, closed arm, and intersection) in order to assess whether a preference towards the open arm developed over repeated testing. Fig 4A–4C show single subject data for percent of total distance travelled in the open arms, percent of total time spent in the open arms and percent of total entries made in to the open arms. Repeated-measures one-way ANOVAs on each dependent measure revealed significant main effects of time for percent open arm distance [F(3.811, 26.68) = 6.726, p = 0.0008], percent open arm time [F(3.681, 25.77) = 5.938, p = 0.0020], and percent open arm entries [F(3.278, 22.95) = 4.307, p = 0.0132]. Table 2 summarizes specific differences between Trial 1 and other trials based on the results of the *post-hoc* analyses. Notably, preference for the open arm defined as exceeding the 50% threshold for percent open arm distance, percent open arm time and percent open arm entries was rare and not stably observed across trials. In agreement with single subject observations, mean values for each measure did not exceed 50% on any trial.

**Table 2:** Statistical results of open arm preference from Experiment 1. Repeated-measures one-way ANOVA and Dunnett’s *post-hoc* test results showing main effects of time across repeated trials for percent of total distance travelled in the open arms, percent of total time spent in the open arms and percent of total entries made to the open arm across 13 EPM trials. Trials not significantly different compared to the first trial are reported as “ns,” and trials that do differ significantly from the first trial are reported with an associated p-value.

		% Open Arm Distance	% Open Arm Time	% Open Arm Entries
**ANOVA p-value**		0.0008	0.0020	0.0132
**Dunnett's Multiple Comparisons**	**1 vs. 2**	ns	ns	ns
	**1 vs. 3**	ns	ns	ns
	**1 vs. 4**	0.0211	ns	0.0178
	**1 vs. 5**	ns	ns	ns
	**1 vs. 6**	ns	ns	ns
	**1 vs. 7**	ns	ns	ns
	**1 vs. 8**	0.0122	0.0170	0.0410
	**1 vs. 9**	ns	ns	ns
	**1 vs. 10**	0.0238	0.0445	ns
	**1 vs. 11**	0.0369	ns	ns
	**1 vs. 12**	ns	ns	ns
	**1 vs. 13**	ns	ns	ns

### Experiment 2: Repeated elevated plus maze trials under distinct lighting conditions

**Fig 5:**
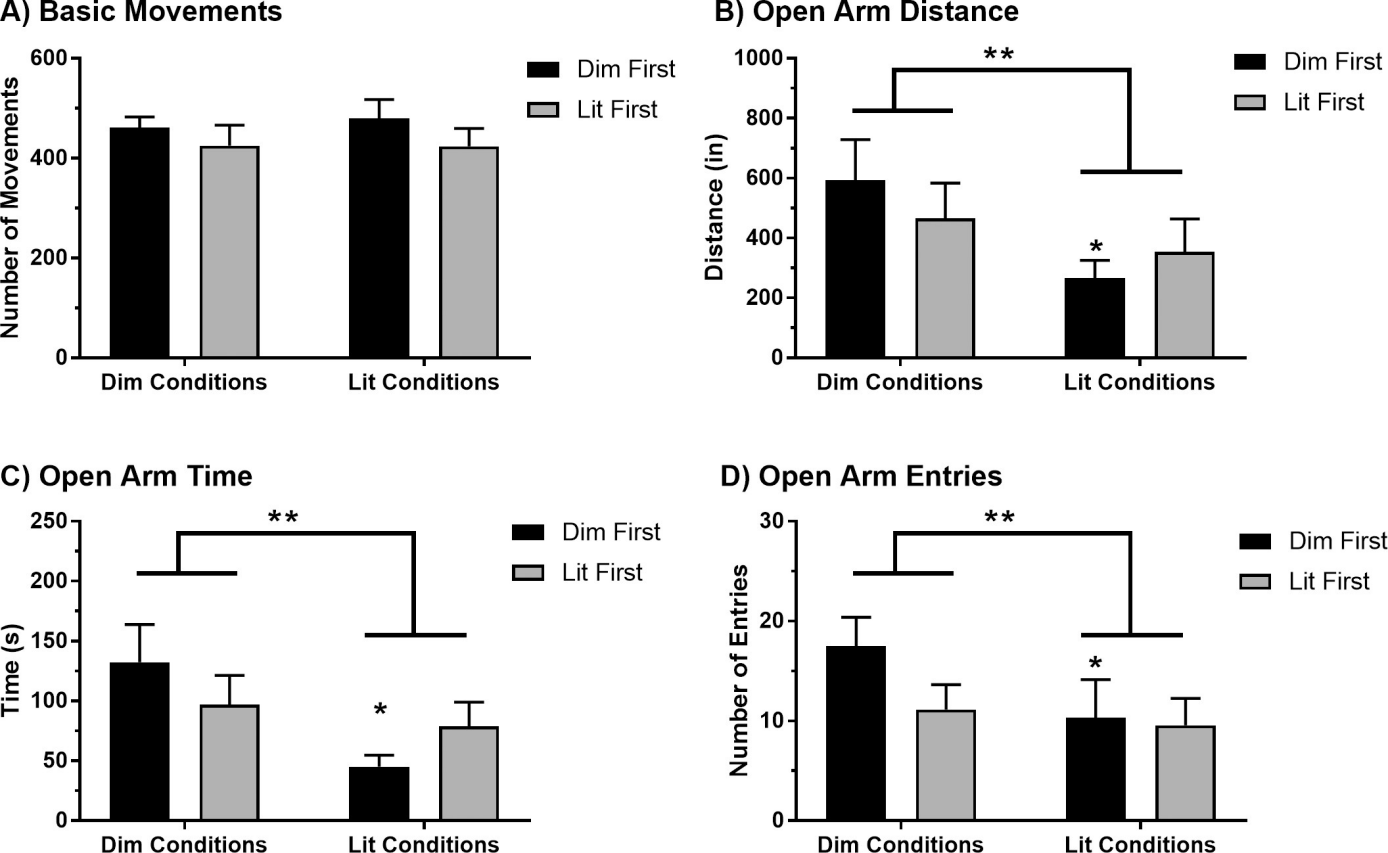
Behavioral performance on the EPM across trials under distinct lighting conditions. Basic movements (A), and open arm distance (B), time (C) and entries (D) across two trials conducted under different lighting conditions. Data are presented as the mean and standard error (Dim First: N = 6; Lit First: N = 7). A significant difference between lighting conditions is denoted by double asterisks (**) and a significant effect of trial order is denoted by a single asterisk (*).

We evaluated behavioral performance across two trials under standard lighting conditions or dim conditions to assess the stability of specific measures across these conditions (see Fig 5). Lighting conditions were counterbalanced, such that one group (Dim First) was evaluated under dim conditions while the other group (Lit First) was evaluated under lit conditions during Trial 1. Conditions were reversed for each group during Trial 2. The results of a repeated-measures two-way ANOVA showed that there was no significant difference in basic movements between the lighting conditions for Trial 1 or 2, regardless of the trial order [main effect of lighting condition, F(1, 11) = 0.236, p = 0.637; main effect of trial order, F(1, 11) = 0.959, p = 0.348; lighting condition x trial order, F(1,11) = 0.299, p = 0.595). For open arm distance and open arm time, rats travelled significantly less [main effect of lighting condition, F(1, 11) = 14.71, p = 0.003; main effect of trial order, F(1, 11) = 0.021, p = 0.888; lighting condition x trial order, F(1, 11) = 3.54, p = 0.087] and spent significantly less time [main effect of lighting condition, F(1, 11) = 11.37, p = 0.006; main effect of trial order, F(1, 11) = 0.00076, p = 0.979; lighting condition x trial order, F(1, 11) = 4.89, p = 0.049] in the open arm under lit conditions compared to dim conditions, but varied depending on the trial order. *Post-hoc* analyses revealed a greater reduction in open arm distance and time under lit conditions compared to dim conditions for rats that were tested under dim conditions for the first trial (distance: p = 0.005; time: p = 0.006). Similarly, the number of open arm entries was significantly lower under lit conditions compared to dim conditions and was also dependent on the trial order [main effect of lighting condition, F(1, 11) = 6.89, p = 0.024; main effect of trial order, F(1, 11) = 0.861, p = 0.373; lighting condition x trial order, F(1, 11) = 2.82, p = 0.121]. *Post-hoc* analyses revealed significantly fewer open arm entries under lit conditions compared to dim conditions for rats that were tested under dim conditions for the first trial (p = 0.027).

## Discussion

The EPM has been used to assess emotional behavior in rodents, most commonly with the limitation that subjects’ performance is evaluated on only one trial [2, 16]. The purpose of our study was to assess behavioral performance over repeated EPM trials to better understand which measures are stable over time and resistant to changes in environmental conditions. Our results show that measures of general behavioral performance (basic movements and total distance travelled) did not differ significantly across repeated trials when compared to the initial trial and did not show changes in linear trends across trials. Likewise, measures of general performance were not altered by experimental lighting conditions. In contrast, differences in measures of open arm avoidance (open/closed arm time, open/closed arm distance, open/closed arm entries) were observed between the first trial and select subsequent trials and significant linear trends indicating decreased open arm avoidance were observed across trials. These observations are consistent with the conclusion that habituation to the open arms of the apparatus occurs with repeated testing. Despite this variability, measures of open arm avoidance were not consistent with preference for the open arm on any measure over repeated trials. Finally, we found that open arm avoidance measures were sensitive to experimental lighting conditions.

The current study shows that measures of general performance are stable across repeated EPM trials, including when lighting conditions are varied. No significant differences in basic movements or its corollary variable total distance travelled were observed over 13 repeated trials or when subjects were tested under standard lighting conditions (in which there is high contrast between the brightly lit open arms and the dimly lit closed arms) or dim lighting conditions (in which there is low contrast between the open and closed arms that are equally lit). The consistency of general performance measures suggests that they are robust outcome variables for detecting emotional behavioral responses to experimental manipulations over time. Our lab previously has shown that exposure to severe stress results in persistent decreases in basic movements on the EPM without changes observed in this measure among non-stressed controls [8, 9, 27]. Specifically, following predator stress, adult rats showed significant decreases in basic movements that were evident at 24-hours post-stress [9]. Repeated testing on the EPM revealed that this stress effect recovered over time but never fully resolved relative to baseline performance across ten trials. Additionally, consistent with the current results, basic movements among non-stressed control subjects were unchanged across the ten trials. This effect appears to be consistent across development as significant decreases in basic movements relative to baseline performance were also observed among adolescent rats following exposure to either predator stress or underwater trauma stress [8]. Interestingly, treatment with a nociceptin/orphanin FQ (NOP) antagonist, which has shown potential use as an anxiolytic, mitigated the behavioral effects in stressed rats without affecting this measure in the non-stressed controls [27]. These studies suggest that deficits in basic movements and related general performance measures following stress may have interpretive value as adverse behavioral responses to stress. The current study reinforces this notion by demonstrating the stability of general performance measures over repeated testing and in changing environmental conditions. Therefore, we postulate that general performance measures can serve as indicators of both locomotor performance and emotional behavioral responses.

In contrast to general performance measures, we observed variability in measures of open arm avoidance across EPM trials. Significant deviations from the initial trial emerged in the fourth trial for the measures of open arm distance travelled and open arm entries and in the eighth trial for open arm time. Each of the deviations reflect reductions in open arm avoidance, perhaps indicative of habituation to the aversive properties of the open arms. These results are consistent with linear trends that show reductions in open arm avoidance across trials. As such, repeated within-subject assessments of open arm avoidance measures can be useful for detecting emotional behavioral responses to experimental manipulations provided that proper controls are included to account for variability across trials and potential habituation to the apparatus. In fact, emotional behavioral responses may become more consistent with experimental manipulation. Indeed, our previous work shows a consistent and persistent decrease relative to baseline in open arm time that was observed among subjects following severe stress exposure [27]. In contrast to stressed counterparts, open arm avoidance behavior of non-stressed controls did not vary significantly over four repeated EPM trials.

To evaluate whether habituation to the open arm substantially reduces the sensitivity of open arm avoidance measures over repeated trials, we expressed each of the measures as percentages of total performance to best detect changes in open arm versus closed arm preference over trials. Consistent with the analysis of the raw data, we found that open arm avoidance was reduced over the course of repeated trials, as indicated by increases in the percent distance travelled, percent time spent and percent of entries made to the open arm in select trials. Notably, open arm avoidance never crossed the threshold for preference on any of the three measures when assessed on the group level, as none of the preference measures exceeded 50%; likewise, single subject data showed that open arm preference measures rarely and inconsistently exceeded the preference threshold. Thus, though subjects do habituate to the open arms of the EPM, the open arm is not preferred even across thirteen trials as assessed by multiple measures of open arm avoidance. Altogether, the consistent biased distribution of behavioral performance for the closed arms relative to the open arms across thirteen trials reveals two key characteristics of the EPM: 1) novelty of the EPM apparatus does not appear to substantially influence relative preference for the open versus closed arms and 2) the sensitivity of open arm avoidance measures is not compromised by repeated testing. Based on these findings, we posit that repeated within-subject EPM testing can be performed and interpreted so long as controls are built in to the experimental and statistical design to account for behavioral variability and open arm habituation across trials.

We found that the lighting conditions in which the EPM test is performed significantly influences open arm avoidance measures but not general performance measures. Likewise, the order in which lighting conditions are experienced influences open arm avoidance measures but not general performance measures. The sensitivity of open arm avoidance measures (and relative resistance of the general performance measures) reflected by these results parallel observations of relative stability of these measures over repeated trials. Specifically with respect to open arm avoidance measures, we found that measures of distance travelled, time spent and entries made to the open arms were all significantly lower when tested under standard lighting conditions relative to dim conditions. Further, this effect was augmented when tested under standard lighting conditions among subjects that had experienced the dim conditions first. Together, these findings show that standard lighting conditions increase open arm avoidance, particularly when subjects have undergone testing in dim conditions for the initial trial. Conditions of higher illumination may increase the aversive properties of the open arms, which appears to have lasting effects given that subsequent testing under dim conditions did not facilitate open arm exploration. In addition to enhancing contrast between the open and closed arms, it is well-documented that a brightly lit environment is in itself aversive to rodents [4]. We postulate that the aversion for the open arms acquired during the initial test carried over to testing in the dim conditions during the second test. This may be evidence of a learned aversion, however further experimentation would be needed to determine this. These results highlight the necessity of consistent experimental conditions over repeated evaluations of behavioral performance on the EPM, especially when assessing measures of open arm avoidance. These results also demonstrate the robust nature of general performance measures on the EPM, underscoring their potential utility in identifying behavioral changes following experimental manipulation.

It is important to note that our study is not designed to specifically address the underlying implications of the one-trial tolerance hypothesis–that the subjective experience that drives behavioral performance on the EPM differs between the initial trial and subsequent trials. Based on the current data, it appears that even if the subjective experience of the initial trial is unique from subsequent trials due to novelty, behavioral output is much the same for at least the initial three trials. Experimental designs could circumvent this issue by habituating subjects to the EPM prior to critical test days or by limiting repeated testing to only a few trials.

Establishing the validity of repeated behavioral testing across time is of great value to neurobehavioral research. Use of within-subject testing affords comprehensive assessment of the effects of experimental manipulations on behavioral performance such that the trajectories of perturbations, treatment and recovery can be tracked with robust experimental design and statistical power. Here, we provide support for the practice of repeated testing on the EPM under selected conditions. We demonstrate that general performance such as basic movements and total distance travelled is robust over repeated trials and experimental conditions. We show that though open arm avoidance habituates over repeated trials and is sensitive to experimental conditions, the open arms retain their aversive properties with repeated testing. Together, these findings suggest that issues of habituation and sensitivity do not preclude the use of repeated testing on the EPM in well-controlled studies and support the value of repeated assessments of behavioral performance on the EPM.
